# Comparative toxicity of five dispersants to coral larvae

**DOI:** 10.1038/s41598-018-20709-2

**Published:** 2018-02-14

**Authors:** A. P. Negri, H. M. Luter, R. Fisher, D. L. Brinkman, P. Irving

**Affiliations:** 1 Australian Institute of Marine Science, Townsville, QLD, and Perth, WA Australia; 2 Australian Maritime Safety Authority, Canberra, ACT Australia

## Abstract

Oil spill responders require information on the absolute and relative toxicities of chemical dispersants to relevant receptor species to assess their use in spill response. However, little toxicity data are available for tropical marine species including reef-building corals. In this study, we experimentally assessed the sub-lethal toxicity of five dispersants to larvae of the coral *Acropora millepora* over three short exposure periods (2, 6 and 24 h) reflecting real-world spill response scenario durations. Inhibition of larval settlement increased rapidly between 2 and 6 h, and was highest at 24 h: EC_50_ Corexit EC9500A = 4.0 mg l^−1^; Ardrox 6120 = 4.0 mg l^−1^; Slickgone LTSW = 2.6 mg L^−1^; Slickgone NS = 11.1 mg L^−1^ and Finasol OSR52 = 3.4 mg L^−1^. Coral larvae were more sensitive to dispersants than most other coral life stages and marine taxa, but the toxic thresholds (EC_10_s) exceeded most realistic environmental dispersant concentrations. Estimating toxic threshold values for effects of dispersants on coral should benefit the decision-making of oil spill responders by contributing to the development of species sensitivity distributions (SSDs) for dispersant toxicity, and by informing net environmental benefit assessment (NEBA) for dispersant use.

## Introduction

Chemical dispersants are used to reduce the impacts of petroleum oil and fuel spills by changing the chemical and physical properties of a surface slick^1^. Typically applied by air or surface vessels, dispersants enhance the mixing of oil into seawater by promoting oil solubility and dilution by forming smaller (less buoyant) oil droplets that become suspended or entrained in the water column. Ultimately the aim is to minimise the impacts of oil slicks on sea life at the water’s surface and the intertidal zone, and to enhance biodegradation^1–3^. The greatest application of dispersants to date was in response to the 2010 Deepwater Horizon wellhead blowout in the Gulf of Mexico, where almost 7 million litres of Corexit EC9500A and Corexit EC9527A were used to disperse slicks both at the surface and by injection at the wellhead, 1500 m below the surface^4^. The influence of this response on the ecological outcomes for shoreline and deep water ecosystems is debated^3,5^, but clearly more studies are needed to test the responses of sensitive biota to oil, dispersed oil and dispersants^6,7^.

Oil spill dispersants usually contain a combination of anionic or non-ionic surfactants with solvents such as glycol ethers and low-aromatic kerosene^8^. Surfactants are critical for enhancing slick dispersal, but they are also considered the more toxic component of dispersant formulations^9^. Surfactants impact organisms in a variety of ways but all are capable of denaturing and binding to proteins and altering the permeability of cell membranes^10^. Contemporary (third generation) dispersants are less toxic than earlier formulations which often contained higher proportions of surfactants or kerosene^9^ and are broadly considered less toxic than aromatic hydrocarbons, the primary contributor to the toxicity of oil and fuel spills^1,11^. The potential hazard posed by each dispersant will depend on the combined effects of the components in each formulation to marine species and this hazard is likely to be species-specific^12,13^.

Chemical dispersal changes the bioavailability of petroleum contaminants in the water column and on the sea floor, in some cases increasing risks to species occupying these habitats^1,14^. It is therefore generally recommended to use dispersants when: (i) the oil is physically and chemically “dispersible” (soon after a spill to maximise efficacy); (ii) the environmental conditions (i.e. depth, currents, and wave energy for mixing) are conducive and (iii) when the application is likely to reduce impacts to surface and shore species, taking into account any subsequent impacts to water column and benthic habitats^1^. Specific guidelines for dispersant use can vary widely; however, there are many situations where the net environmental benefits of chemical dispersion are not clear^15,16^. For example, a slick in close proximity to both a coral reef and a mangrove habitat may present the need to choose between the risks posed by a predominantly floating slick (which may persist in a mangrove system for many years) and dispersant use that increases the immediate, acute exposure of the sub-surface corals^17,18^. Resolving this issue requires information on the expected exposure type and duration as well as the hazards posed by oil, chemically dispersed oil and the dispersants themselves. If chemical dispersal of a spill is considered to provide a net environmental benefit, then the comparative efficacy, cost and potential toxicity of dispersants to sensitive “receptor” species should also be considered^19^.

Oil and gas extraction often occurs near coral reefs in the Middle East, throughout South East Asia, in the Caribbean and across north-western Australia and the Timor Sea, and shipping traffic continues to increase in close proximity to coral reefs, including the Great Barrier Reef^20^. While these activities continue, there is always a low likelihood but potentially catastrophic risk posed by uncontrolled spills to tropical coral reefs. It is generally not recommended to apply dispersants in shallow environments or near coral reefs^1,17^; however, dispersants were employed to reduce the surface slicks during the grounding of the *Shen Neng* 1 on a reef in the Great Barrier Reef Marine Park^21,22^. A range of six different dispersants (totalling 184 m^3^)^23,24^ were also applied in response to the estimated 4700 m^3^ medium crude spill from the Montara well head platform off tropical north Western Australia to minimise the risks of surface slicks reaching coral reefs^23^. The toxicity of oil and/or chemically dispersed oil to corals has been summarised and reviewed recently^15,25,26^. Although it is difficult to compare toxicities between studies due to inconsistencies in methodologies and toxicity metrics, in general, early life phases of corals are more sensitive to hydrocarbons and dispersants than adults. Table 1 summarises the toxicity of dispersants alone to various life phases of coral available from eight publications since 2000. While many of the dispersant formulations were not toxic to adult coral branches at concentrations of 10 mg l^−1^, concentrations below this often affected the survival and settlement of larvae for some species. The Australian National Plan benchmark for acceptable toxicity is based on a multiple day exposure to an EC_50_ or equivalent, depending on species and test^27^.

**Table 1:** Summary of published dispersant toxicity studies to different life stages of corals since 2000. EC_50_ where 50% organisms are affected. NOEC = no observed effect concentration. LOEC = lowest observed effect concentration. NA = affected at the lowest concentration tested.

Dispersant	Species	Life stage	Endpoint	Reported toxicities (mg/L)	Reference
Corexit EC9500A	*Porites astreoides*	Larvae	Settlement 48 h	NA (NOEC), 25 (LOEC)	^58^
	*Porites astreoides*		Mortality 72 h	25 (NOEC), 50 (LOEC)	^58^
	*Montastraea faveolata*		Settlement 48 h	NA (NOEC), 25 (LOEC)	^58^
	*Montastraea faveolata*		Mortality 72 h	NA (NOEC), 25 (LOEC)	^58^
	*Xenia elongata* ^*a*^	Adult	Bleaching 24 & 72 h	NA (NOEC), 5 (LOEC)	^34^
	*Swiftia exserta* ^*a*^		Health^b^ 48 h	64 (LC_10_), 70 (LC_50_)	^33^
	*Paramuricea sp*.		Health^b^ 96 h	NA (NOEC), 35 (LOEC)	^59^
	*Callogorgia delta*		Health^b^ 96 h	NA (NOEC), 35 (LOEC)	^59^
	*Leiopathes glaberrima*		Health^b^ 96 h	NA (NOEC), 35 (LOEC)	^59^
Corexit EC9527A	*Acropora millepora*	Gametes	Fertilisation, 4 h	1 (NOEC), 10 (LOEC)	^49^
	*Acropora millepora*		Settlement, 24 h	1 (NOEC), 5 (LOEC)	^49^
	*Montastraea franksi*	Adult	Gene expression^c^, 8 h	1 (NOEC), 5 (LOEC)	^60^
Slickgone NS	*Stylophora pistillata*	Adult	Mortality^d^ 24 h	125 (NOEC), 250 (LOEC)*	^61^
	*Pocillopora damicornis*		Mortality^d^ 24 h	25 (NOEC), 50 (LOEC)*	^61^
Dispolen 36S	*Stylophora pistillata*	Adult	Mortality^d^ 24 h	50 (NOEC), 125 (LOEC)*	^61^
	*Pocillopora damicornis*		Mortality^d^ 24 h	25 (NOEC), 50 (LOEC)*	^61^
Inipol 90	*Stylophora pistillata*	Larvae	Mortality 96 h	5 (NOEC), 50 (LOEC)*	^62^
	*Stylophora pistillata*		Mortality 96 h	0.5 (NOEC), 5 (LOEC)*	^62^
	*Stylophora pistillata*	Adult	Mortality^d^ 24 h	50 (NOEC), 125 (LOEC)*	^61^
	*Pocillopora damicornis*		Mortality^d^ 24 h	50 (NOEC), 125 (LOEC)*	^61^
Petrotech PTI-25	*Stylophora pistillata*	Larvae	Mortality 96 h	5 (NOEC), 50 (LOEC)*	^62^
	*Stylophora pistillata*		Settlement 96 h	NA (NOEC), 0.5 (LOEC)*	^62^
	*Stylophora pistillata*	Adult	Mortality^d^ 24 h	50 (NOEC), 125 (LOEC)*	^61^
	*Pocillopora damicornis*		Mortality^d^ 24 h	125 (NOEC), 250 (LOEC)*	^61^
Bioreico R-93	*Stylophora pistillata*	Larvae	Mortality 96 h	5 (NOEC), 50 (LOEC)*	^62^
	*Stylophora pistillata*		Settlement 96 h	NA (NOEC), 0.5 (LOEC)*	^62^
	*Stylophora pistillata*	Adult	Mortality^d^ 24 h	50 (NOEC), 125 (LOEC)*	^61^
	*Pocillopora damicornis*		Mortality^d^ 24 h	25 (NOEC), 50 (LOEC)*	^61^
Emulgal	*Stylophora pistillata*	Larvae	Mortality 96 h	5 (NOEC), 50 (LOEC)*	^62^
	*Stylophora pistillata*		Settlement 96 h	NA (NOEC), 0.5 (LOEC)*	^62^
	*Stylophora pistillata*	Adult	Mortality^d^ 24 h	50 (NOEC), 125 (LOEC)*	^61^
	*Pocillopora damicornis*		Mortality^d^ 24 h	25 (NOEC), 50 (LOEC)*	^61^
Biosolve	*Stylophora pistillata*	Larvae	Survivorship 96 h	5 (NOEC), 50 (LOEC)*	^62^
	*Stylophora pistillata*		Settlement 96 h	NA (NOEC), 0.5 (LOEC)*	^62^

*Assuming ~500 mg l^−1^ stock.

^a^Octocorals.

^b^Branch health 96 h incorporated partial mortality and other stress responses.

^c^HSP70 expression upregulation. HSP90 and P-GP were up-regulated at 10 mg l^−1^.

^d^Survival one week after a 24 h exposure.

Surface oil slicks respond best to chemical dispersion prior to weathering changing the composition of the slick. Dispersion becomes less effective within hours to days, depending on the oil and conditions^9^. In most spill scenarios, exposures of marine organisms to peak concentrations of hydrocarbons and dispersants (when used) is expected to be short^23,28^ (minutes to hours), even when there is a prolonged discharge, because local marine and weather conditions dilute the potential toxicants through mixing, currents and tides. Therefore, it has been proposed that environmentally relevant toxicity tests should consider shorter exposures than the standard multiple day laboratory toxicity protocols^14,15,19^. In this study the toxicity of five dispersant formulations to coral larvae were tested over three short exposure periods (2, 6 and 24 h). The inhibition of larval settlement was selected as a potentially sensitive and ecologically relevant endpoint and we interpolated the toxicity thresholds (EC_10_ and EC_50_, where 10% and 50% of settlement is inhibited, respectively) from concentration-response curves^29^.

## Results

**Figure 1:**
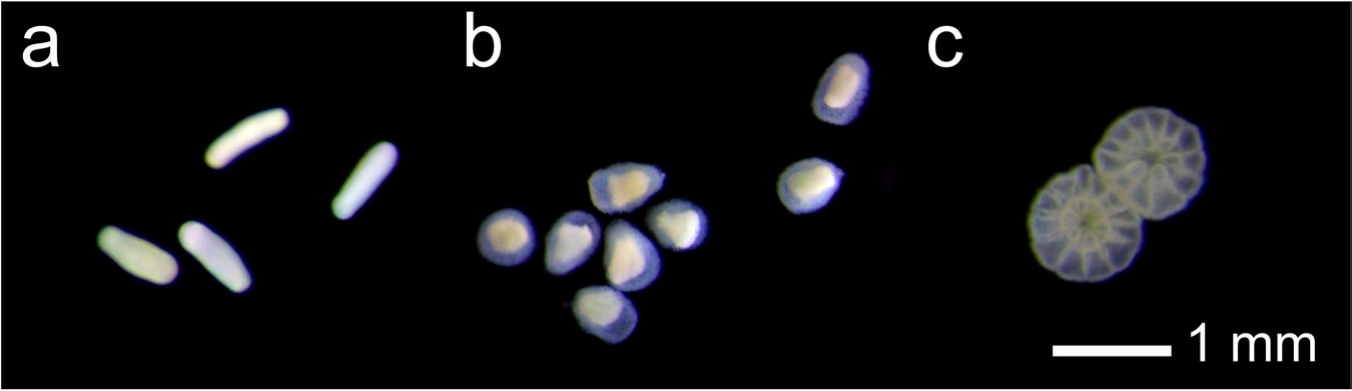
Photomicrographs of 7 d-old *A*. *millepora* larvae following 24 h exposure to (**a**) control seawater and (**b**) 10 mg l^−1^ Slickgone LTSW. Normal post-settlement metamorphosis of control larvae after an additional 18 h (**c**), with mesentery and tentacle formation evident in the single polyps.

In uncontaminated water (control treatments) and in the presence of the lowest concentrations of dispersant, ~1 mm long larvae were typically cigar-shaped and highly motile (Fig. 1a). As the concentration and exposure time increased (for all dispersants) larvae became less motile, forming a squat, bullet shape with ill-defined and transparent outer membranes (Fig. 1b). Similar effects were observed for the reference toxicant SDS. After exposure to uncontaminated seawater (2, 6 and 24 h) and following the addition of chemical inducer, 85–95% of control coral larvae underwent successful attachment and metamorphosis into single polyp juveniles (Fig. 1c, Table 2).

**Table 2:** Metamorphosis success (% ± SE, n = 6) of *A. millepora* larvae after 2, 6 and 24 h exposure to filtered seawater (control treatments only).

Dispersant treatments	% metamorphosis controls		
	2 h	6 h	24 h
Ardrox 6120	95 ± 3	85 ± 4	89 ± 5
Corexit EC9500A	94 ± 2	86 ± 5	89 ± 4
Slickgone LTSW	90 ± 5	87 ± 5	87 ± 4
Slickgone NS	90 ± 4	86 ± 6	93 ± 2
Finasol OSR 52	85 ± 5	88 ± 5	93 ± 3
SDS	85 ± 7	93 ± 3	90 ± 3

**Figure 2:**
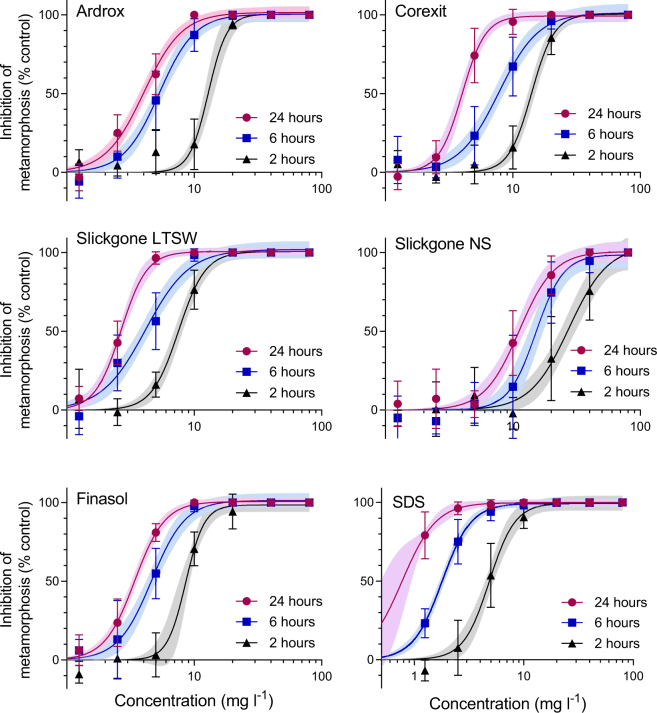
Concentration response curves for inhibition of metamorphosis of *A. millepora* larvae by dispersants measured following 2, 6 and 24 h exposures. Bars represent SD ± n = 6. Shaded areas represent the 95% confidence boundaries of each 4 parameter sigmoidal model. All effect concentrations (EC_10_ and EC_50_) for each dispersant at each time point are provided in Table 3.

The effects of exposure to all dispersants and SDS followed typical concentration-response curves with increasing concentrations (Fig. 2). Fitting the % inhibition data to 4-parameter sigmoidal equations (R^2^ values 0.80–0.98, Table 3) enabled the interpolation of concentrations that inhibited metamorphosis in *A. millepora* larvae by 10% (EC_10_) and 50% (EC_50_) (Table 3). Of the five dispersants, Slickgone LTSW was the most toxic (24 h EC_50_ = 2.6 mg l^−1^), while Slickgone NS was the least toxic (24 h EC_50_ = 11.1 mg l^−1^). Exposure to the lowest concentration of SDS (1.25 mg l^−1^) at 24 h caused 79% inhibition so EC_XX_ values for SDS were not reliable at this time point.

**Table 3:** Concentrations of dispersants that inhibited the metamorphosis success of *A. millepora* larvae by 50% and 10% (EC_50_ and EC_10_), over three exposure periods and calculated from concentration-response curves presented in Fig. 2. 95% confidence intervals in parentheses. F-test results demonstrating the significant effect of exposure duration on EC_50_ values. PC95 = 95% species protection values (=HC5) derived from species sensitivity distributions for Ardrox, Slickgone LTSW, Slickgone NS, Finasol^36^ and Corexit EC9500A^37^.

Dispersant	EC_50_ (mg l^−1^)			EC_10_ (mg l^−1^)			P	PC95 (mg l^−1^)
	2 h	6 h	24 h	2 h	6 h	24 h	F(df)	(No. species)
Ardrox 6120	12.8	5.3	4.0	8.9	2.6	1.9	<0.0001	0.013
	*(11.8*–*14.4)*	*(4.8–5.9)*	*(3.6–4.3)*	*(8.0–9.9)*	*(2.1–3.2)*	*(1.6–2.3)*	157 (2, 138)	(3)
Corexit C9500A	14.0	7.7	4.0	8.9	3.5	2.5	<0.0001	6.6
	*(13.0–15.1)*	*(6.8–8.6)*	*(3.7–4.3)*	*(8.0–10.1)*	*(2.8–4.5)*	*(2.2–3.0)*	196 (2, 138)	(18)
Slickgone LTSW	7.5	4.1	2.6	4.4	1.7	1.6	<0.0001	0.017
	*(6.8–8.2)*	*(3.3–5.1)*	*(2.5–2.8)*	*(3.8–5.2)*	*(1.1–2.5)*	*(1.4–1.8)*	80.1 (2, 138)	(4)
Slickgone NS	26.4	15.4	11.1	12.9	8.8	5.6	<0.0001	0.1
	*(22.7–30.9)*	*(13.1–17.7)*	*(10.0–12.5)*	*(9.9–17.1)*	*(6.9–11.5)*	*(4.5–7.1)*	37.2 (2, 138)	(7)
Finasol OSR 52	8.6	4.6	3.4	5.9	2.4	1.9	<0.0001	0.13
	*(7.5–9.3)*	*(4.1–5.1)*	*(3.2–3.6)*	*(4.7–7.4)*	*(1.9–3.0)*	*(1.7–2.1)*	122 (2, 138)	(7)
SDS	4.9	1.8	0.77	2.7	0.91	0.35	<0.0001	—
	*(4.4–5.4)*	*(1.7–1.9)*	*(0.48–0.95)*	*(2.1–3.4)*	*(0.80–1.0)*	*(0.20–0.65)*	195 (2, 135)	

**Figure 3:**
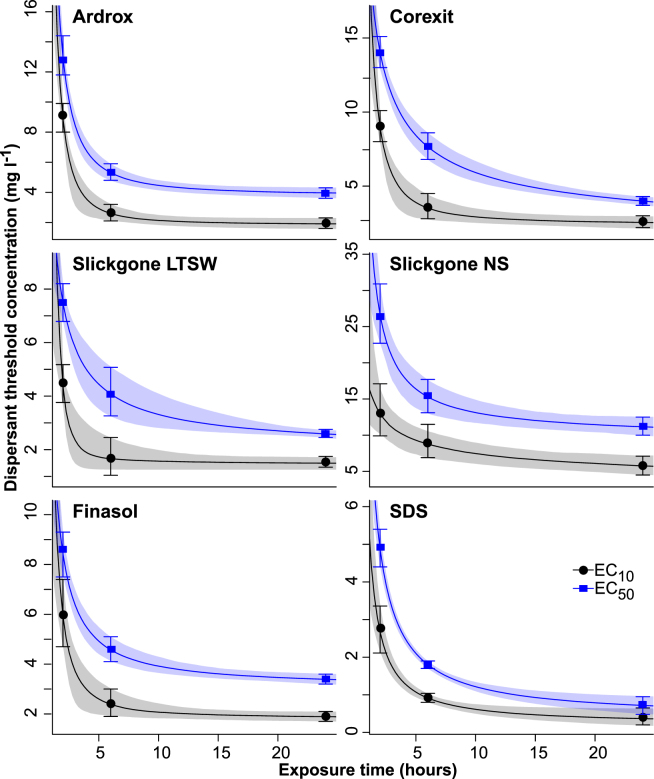
Time dependence of estimated EC_10_ and EC_50_ concentrations for each dispersant, with values and 95% confidence bars taken from Table 4. Solids lines are fitted three parameter exponential decay relationships, with shaded areas representing 95% confidence bands based on 1000 iterations of a monte-carlo simulation sampling from gamma distributions fitted to the estimated threshold concentration values and their 95% confidence bands.

The exposure duration had a strong effect on the toxicity of all five dispersants and SDS to coral larvae, with EC_50_ values decreasing by an average 2.9-fold as the exposure duration increased from 2 to 24 h (Table 3). The EC_50_ for SDS also decreased dramatically as the duration of exposure increased from 2 h to 6 h (Table 3). F-test comparisons from the fitted curves demonstrated a significant effect of exposure duration on EC_50_ values for each dispersant and SDS (Table 3). The time-dependent relationship between the EC_x_ values and concentration was captured using a three parameter exponential decay function (Fig. 3). The EC_x_ values can be predicted for other exposure times using Equation 1 with the estimated parameters in Table 4.

**Table 4:** Parameter estimates for the time-dependence of EC_10_ and EC_50_ concentrations for each dispersant for the three parameter exponential decay function *(y* = *m* + *a* * *exp*(−*b* * *log*(*x*)) where *y* is the EC_10_ or EC_50_ threshold concentration, *x* is exposure time in hours, *exp(*−*b*  * *log(x))* is the exponential function, *m* is the concentration at which an infinite exposure time would theoretically yield no effect, *a* is the theoretical threshold concentration at zero exposure (y-intercept) and *b* is the exponential rate of decay. 95% confidence intervals in parentheses.

Dispersant	EC_50_ curve parameters			EC_10_ curve parameters		
	a	b	m	a	b	m
Ardrox 6120	29.9	1.67	3.79	40.2	2.22	1.85
	*(20.3–43.2)*	*(1.21–2.23)*	*(3.3–4.25)*	*(17.5–182)*	*(1.38–4.82)*	*(1.42–2.29)*
Corexit EC9500A	20.6	0.644	0.698	27.3	1.76)	2.35
	*(18–24.4)*	*(0.275–1.02)*	*(−5.67–3.19)*	*(12.3–97.3)*	*(0.832–3.84*	*(1.47–2.9)*
Slickgone LTSW	12	0.899	0.835	82.7	3.61	1.48
	*(8.6–26.3)*	*(0.0985–1.8)*	*(−14.7–2.63)*	*(5.63–320)*	*(0.793–6.71)*	*(1.12–1.75)*
Slickgone NS	38.5	1.06	8.29	39.5	0.561	−18.7
	*(24.1–70)*	*(0.281–2.03)*	*(−2.79–12.2)*	*(10.5–137)*	*(0.0208–2.08)*	*(−113–6.74)*
Finasol OSR 52	12.4	1.18	3.02	23.3	2.06	1.81
	*(8.62–18.2)*	*(0.653–1.77)*	*(2.13–3.48)*	*(7.22–128)*	*(0.911–4.98)*	*(1.4–2.08)*
SDS	9.43	1.06	0.384	5.83	1.2	0.219
	*(7.72–11.6)*	*(0.823–1.36)*	*(−0.0524–0.808)*	*(3.74–9.47)*	*(0.711–1.92)*	*(−0.196–0.6)*

## Discussion

Coral larval settlement was sensitive to acute exposures to all five individual oil spill dispersants formulations, with the threshold concentrations for toxicity (EC_10_s) all relatively low in comparison with other coral life stages and marine taxa. The toxicity of each dispersant increased with exposure duration until 6 h, indicating rapid uptake of the toxic components of the formulations and this could be related to the small size and high surface area  to volume of the larvae. Although the toxic thresholds were low, they were higher than dispersant concentrations that would be expected in the real-world following applications in tropical waters. Differences in the toxicity observed between dispersants was likely due to the combined toxic effects of different formulation components and their relative concentrations. The toxic threshold values determined here for each dispersant should contribute to the development of species sensitivity distributions (SSDs) for dispersant toxicity and support net environmental benefit assessments (NEBAs) for dispersant applications, to improve decision making by oil spill responders.

### Comparative toxicity between dispersants

All of the dispersant formulations were observed to affect larvae in a similar way. Settlement and metamorphosis was reduced at low concentrations, while impaired mobility, changes to larval shape and increased membrane transparency were typically observed at higher concentrations. Although each dispersant formulation is different, similar toxic modes of action between formulations might be expected as all contain the anionic surfactant DOSS, which is likely to be a major contributor to toxicity^9,15,30^. The reference toxicant SDS caused a similar response pattern and (like DOSS) was likely to have damaged membrane integrity, leading to a failure of cellular homeostasis^30^. Additional impacts on membrane proteins, lipopolysaccharides and other cellular functions cannot be discounted^31^. Surfactants have a wide range of toxicities, with no effect concentrations of environmentally relevant endpoints reported between 0.01 and 100 mg l^−1^, and sub-lethal responses often occurring at concentrations an order of magnitude lower^32^. The 24 h thresholds for toxicity (EC_10_) of between 1.6–5.6 mg l^−1^ for the five dispersants were similar to the toxicities of other dispersants to coral larvae summarised in Table 1, but direct comparisons with these other studies are difficult as EC_10_s have not previously been calculated for corals from concentration response curves. The only other study to publish EC_10_ or EC_50_ values on the effects of dispersants to coral, indicated that Corexit EC9500A was more than 10-fold less toxic to adult fragments of a temperate octocoral^33^ than to *A. millepora* larvae. The current study allowed direct comparisons of toxicity between the five dispersants to the same species under identical conditions. This matched dataset indicated that although there were differences in toxicity between the dispersants to coral larvae, the range of EC_50_s was relatively narrow (2.6 and 11 mg l^−1^) after 24 h. The acute toxicity of each formulation should be linked to the mixture toxicity (combined potency and concentration of surfactants and other components) of the dissolved fraction^30^, but without detailed chemical analysis (not available here), assigning  specific reasons for these differences would be speculative.

### Time-dependent toxicity

To examine the time-dependency of toxicity, coral larvae were exposed to dispersants over three short periods representing short peaks in concentration expected during an oil spill response^19,28^. There was a rapid exponential change in toxicity between 2 and 24 h with the majority of the ~3-fold increase occurring between 2 and 6 hours (Fig. 3). The minor changes in toxicity between 6 and 24 h exposures indicate that maximum acute toxicity to the small coral larvae occurred rapidly for all of the dispersants tested. In adult soft corals, Corexit EC9500A exposure caused bleaching (the loss of symbionts) but no time-dependent effects were observed between 24 and 72 h^34^. The rapid onset of toxicity for larvae could be related to their small size and high surface area to mass ratio, leading to fast uptake kinetics^35^. The acute toxicity results reported here are relevant to short-term field exposures expected in the majority of response scenarios where applications are short or tides and currents reduce exposure from longer applications^23,28^. The consistent exposures applied over three short periods were followed by settlement in uncontaminated water. Additional short and long term exposures tests should be conducted to assess a variety of other exposure scenarios. Acute exposures of larvae to spikes of dispersant followed by gradual dilution during settlement is a more likely natural scenario and should be tested, but deriving effect thresholds for comparison with other studies is more difficult. Extended low concentration exposure tests should also be conducted to assess the potential chronic effects posed by longer term dispersant responses^4^.

### Toxicity relative to regulatory values

Ideally, responders to an oil spill would know the relative toxicity of each available dispersant in combination with the specific oil type for species relevant to the spill site^19^. Since these data are rarely available, the comparative toxicity of dispersant formulations to the relevant taxa (as measured here for corals) may be valuable for combination with modelled concentrations to assess risk and to help choose the least harmful dispersant for the response. Each national or state jurisdiction has its own regulations or guidelines on the appropriate use of dispersants. For example, some European nations require toxicity tests to demonstrate that dispersant formulations are less toxic than a reference toxicant or do not enhance the toxicity of oil in comparison to mechanically dispersed oil^17^. Guidelines set by the Australian Maritime Safety Authority (AMSA) under the National Plan limit the acceptability for use of dispersant products that show a toxic effect (EC_50_, LC_50_ or similar) to a diversity of marine species, including microalgae, copepods, amphipods, sea urchins, scallops and fish, at concentrations of 10 mg l^−1^ or less^27^. Inhibition of larval settlement and metamorphosis is considered an ecologically relevant endpoint for water quality derivation^29^. If the 24 h coral larval exposure results (Table 3) were to be included within this AMSA multi-taxa approach, then only Slickgone NS would have met this requirement.

Another approach that should be considered to compare and regulate dispersant safety involves the application of species sensitivity distributions (SSD); probability models of the sensitivity of multiple taxa to dispersant exposure. Adams^36^ recently developed preliminary SSDs for a range of dispersants registered for use in Australia based on the very limited set of toxicity data available for marine species. The SSDs were used to determine the predicted no-effect concentration which was assigned as 95% species protection (PC95, this is equivalent to the HC5 where 5% of species are affected). The PC95s (Table 3), were generally derived from chronic EC_10_ values and were of low to moderate quality due to the lack of toxicity data (only 3 to 7 species available per dispersant). More toxicity data is available for Corexit EC9500A and a PC95 of 6.6 mg l^−1^ was derived by Barron *et al*.^37^. A comparison of the acute EC_10_s for coral larval settlement with the sensitivity data reported by Adams^36^ and reviewed by Hook and Lee^15^, indicates that coral larvae are relatively sensitive to Ardrox, Slickgone LTSW, Slickgone NS and Finasol compared with many other species tested, especially if the acute toxicity thresholds derived here were converted to chronic values (by dividing by a factor of 2–5)^29^. However, the PC95 values derived from limited toxicity data would be broadly protective of coral larvae to these dispersants. Coral larvae were also relatively sensitive to Corexit EC9500A in comparison to many other fish, mollusc and crustacean species^37,38^ but would not be protected by the PC95 derived by Barron *et al*.^37^.

When a NEBA is undertaken to assess whether to apply dispersants to help degrade oil slicks and protect intertidal and shoreline habitats, it requires input of a very broad range of information that includes the type and size of a spill, weather conditions, hydrographic and oceanographic conditions, the relative presence and distribution of vulnerable receptors to both dispersed and undispersed oil, accessibility and availability of application, and monitoring resources and predictive modelling^9,15^. Dispersant application may increase the exposure of sub-surface species to greater doses of petroleum hydrocarbons, and this additional exposure and potential harm from dispersants themselves is also a critical consideration for the NEBA process^19^. While specific studies should continue to be conducted to compare the toxicity of physically versus chemically dispersed oil, the current study demonstrated that these five dispersants were approximately an order of magnitude less toxic to coral larvae than dissolved aromatic hydrocarbons from a water accommodated fraction of light crude oil in very similar tests^25^. This is consistent with the general notion that dispersants alone are less toxic than oil, but chemically dispersed oil is more toxic due to greater concentrations of oil in water^5,12,39^.

Comparing toxic thresholds for dispersants derived from laboratory studies with concentrations measured in the field are particularly difficult as dispersant formulations are comprised of multiple potentially toxic components. These components are likely to retain their original proportions in short acute experiments, but these proportions will change rapidly when formulations are applied in the field. Field and laboratory comparisons are further complicated as concentrations can be expressed as dilutions of the formulation (which can only be modelled in the field) or as concentrations of marker/proxy components, such as DOSS (which doesn’t account for different behaviour and toxicity of other components). The highest concentration of Corexit EC9500A at the water surface soon after application by air (dispersant:oil ratio 1:20) has been estimated as ~5 mg l^−1^^40^ and up to 13 mg l^−1^ of dispersant measured in sea trials^41^. The EC_10_ thresholds for 24 h exposure of coral larvae to dispersants were 1.6–5.6 mg l^−1^, indicating that coral larvae in the immediate vicinity of dispersant application could be at risk. However, rapid dilution at sea is expected to reduce exposure concentrations^1,28,41^. For example, measured concentrations of DOSS as a presumed marker for Corexit EC9500A and EC9527A (since other sources of DOSS were identified in the Gulf^42,43^) reported in weeks following the Deepwater Horizon spill were far less than the EC_10_ values for dispersant formulations reported here for coral larval settlement^44,45^. Nevertheless, more tropical species toxicity data, especially for sensitive reproductive stages of reef-building corals and for relevant exposure durations, are crucial to improve the SSD approach to assess environmental safety and to contribute to a more robust information base for the NEBA process and decision support for oil responders^15,16,36,37^. Other operational considerations reinforce this conclusion. For example, Australian dispersant operations protocols recommend that dispersants not be applied without sufficient depth of water, or water exchange, or near coral reefs^1,9,46^. The most sensitive reproductive processes for many reef-building corals occur during and soon after discrete mass spawning events that usually occur annually at a given reef, and where spawn and larvae are present in the water column between reefs for days to weeks^47^. This limits the likelihood of exposing mobile coral larvae to oil spills for most of the year, but dispersant applications during the annual coral spawning seasons (even long distances from coral reefs) should be carefully considered in the NEBA process.

## Materials and Methods

Coral larvae were exposed to eight concentrations of five different dispersants for durations of 2, 6 or 24 h. After these exposure periods the larvae were rinsed and the larvae allowed to settle and undergo metamorphosis over an additional 18 h in uncontaminated seawater. The concentrations of dispersant that inhibited metamorphosis by 10% and 50% (EC_10_ and EC_50_) were calculated from concentration-response curves for each exposure duration.

### Coral collection and larval culture

Colonies of the common Indo-Pacific broadcast spawning coral *Acropora millepora* (Ehrenberg, 1834) >20 cm were collected from ~3 m depth in October 2015 from Esk Island, on the central Great Barrier Reef (GBR, 18°46.420′S 146°31.372′E). This species has been used in similar larval assays for over a decade and has predictable spawning and settlement behaviour^48–52^. Gravid colonies were transported to the National Sea Simulator (SeaSim) at the Australian Institute of Marine Science (Townsville, Australia) and placed in flow-through tanks at ~27 °C until spawning. Gametes were collected from seven parental colonies on a single night, fertilized and the symbiont-free larvae were cultured at less than 500 larvae l^−1^ in flow through tanks as previously described^49^. *A. millepora* larvae reach maximum competency for settlement after six days (reviewed in Jones *et al*.^47^) and seven day old larvae were used in these exposure experiments.

### Dispersant preparation

Dispersants (Table 5) were provided by AMSA. The dispersants chosen were those listed at the time on the Australian National Plan Register of Oil Spill Control Agents and so available for deployment in an oil spill. Subsequently, Dasic Slickgone LTSW and Ardrox 6120 have been removed from operational stockpiles as obsolete stock. Concentrated stock solutions (4 g l^−1^) of each dispersant were prepared in 1 µm filtered seawater (FSW) and the homogeneity of stocks was maintained by rapidly stirring with a Teflon-coated magnetic bar. Solutions of eight nominal concentrations (0, 1.25, 2.5, 5, 10, 20, 40 and 80 mg l^−1^) were prepared by dilution of the stirring stock with FSW and each of these exposure solutions were used within 2 h of preparation. Nominal concentrations of each formulation were reported in the experiment as the more toxic components, including DOSS, are water soluble and little degradation and loss of volatiles would occur over the short assay periods. The alternative (using measured concentrations) would require estimation of potential dispersant losses from concentrations of a proxy component, and this could be misleading in the case of the complex dispersant formulations where minor components may behave differently and/or contribute more or less toxicity than the measured component. Furthermore, direct comparisons between the toxicity of distinct dispersant formulations are also assisted by expressing the treatments in mg (formulation) l^−1^. Sodium dodecyl sulphate (SDS), an anionic surfactant, was prepared in the same way and used as a reference toxicant.

**Table 5:** Tested dispersants and the hazardous constituents listed in their safety data sheets; percentages are included, where available. DOSS (dioctyl sodium succinate) is an anionic surfactant and CXX denotes the carbon number of hydrocarbons (HCs).

Dispersant	Hazardous Constituents Listed in Safety Data Sheet	CAS No.
Corexit EC9500A	10–30% DOSS	577-11-7
	10–30% hydrotreated petroleum distillate	64742-47-8
	1–5% 1,2-propanediol	57-55-6
Finasol OSR 52	20–25% DOSS	577-11-7
	15–20% C11-C14 HCs (<2% aromatics)	926-141-6
	15–20% (2-methoxymethylethoxy)propanol	34590-94-8
Slickgone NS	DOSS (unspecified %)	577-11-7
	hydrotreated petroleum distillate (unspecified %)	64742-47-8
Slickgone LTSW	10–20% DOSS	577-11-7
	30–40% 2-butoxyethanol	111-76-2
Ardrox 6120	10–30% Anionic surfactants (including soaps)	mixture
	10–30% 2-butoxyethanol	111-76-2

### Settlement assays

Coral larvae were statically exposed in acetone-rinsed 20 ml glass vials containing 10–12 coral larvae and 15 ml dispersant solutions with six replicate vials used for each dispersant concentration. Vials were sealed with aluminium-lined caps and a ~7 ml headspace allowed oxygen exchange. Vials were then transferred to an orbital incubator/shaker (50 rpm) to maintain gentle water movement and to discourage settlement^25^. Temperature was set at 27 °C and light at 40 µmol quanta m^−2^ s^−1^. Under these conditions dissolved oxygen was maintained at >7 mg l^−1^, and pH (8.0–8.2) and salinity 33–35 psu (Table S-1, Supplementary information). Three exposure durations were tested with vials removed from the incubator after 2, 6 or 24 h exposures. To enable the testing of multiple treatments, the start times for each group of exposure durations (24 h, 6 h and 2 h in sequence) were offset by 2 h. Control (uncontaminated seawater) exposures were conducted (n = 6) for each dispersant type at each time point. Following the exposures, larvae were transferred into small (15 mm wide) 100 µm nylon mesh filters which were partially submerged and the larvae gently washed with an excess of uncontaminated FSW. The dispersant-free larvae were then transferred into individual 6-well cell culture plates containing 10 ml FSW (12 ml, Nunc, NY, USA).

Settlement and metamorphosis of coral larvae in the plastic well plates was initiated by the addition of a slightly sub-optimal (to maximise the sensitivity of the assay) concentration (5 µl) of crustose coralline algae (CCA) extract^51^ prepared using 4 g of the crustose coralline algae *Porolithon onkodes*^48^. Following exposure the settlement inducer, larvae cease swimming within minutes and elongate before undergoing early metamorphosis within 12 h^48,53^. Metamorphosis was assessed after 18 h and larvae scored as normal and functional if they had changed from either the free swimming or casually attached pear-shaped forms to squat, firmly attached, disc-shaped structures with pronounced flattening of the oral–aboral axis and with septal mesenteries radiating from the central mouth region^48^. This assay therefore assessed whether larvae were functional following the dispersant exposure. Larval settlement above 70% in the controls was considered acceptable as an endpoint based upon several previous studies using CCA or extracts of CCA to initiate settlement of *Acropora* spp.^25,54–56^.

### Data analysis

Inhibition of metamorphosis (% inhibition relative to 0% WAF control) was calculated from treatment data as Inhibition (%) = 100 × [(% metamorphosis_control_ − % metamorphosis_treatment_)/% metamorphosis_control_]. The concentration of dispersant that inhibited 10% and 50% of metamorphosis (EC_10_ and EC_50_) was calculated from concentration-response curves (four-parameter sigmoidal models) fitted to the % inhibition and total aromatics data of each treatment using the program GraphPad Prism (v7, San Diego, USA). The probability that EC_50_ values generated by the logistic curves were statistically different was tested by applying the F test in Graph Pad Prism v6. EC_50_ values were considered different when p < 0.05.

A Monte Carlo approach was carried out using the R software^57^ to propagate uncertainty in the EC_10_ and EC_50_ thresholds (calculated independently for each experimental exposure time) and to provide parametric model fits (and associated uncertainty) for estimating thresholds as a continuous function of exposure time that might be more readily incorporated into oil spill modelling. This was achieved in two steps: (i) a gamma distribution was fitted to the mean, lower and upper confidence bands for the EC_10_ and EC_50_ estimated threshold concentrations for each experimental time; (ii) secondly 1000 random new concentration values were generated using these fitted distributions for each of the three exposure times, and fitted these using a three parameter exponential decay function:

1 $$y=m+{a}^{\ast }exp(-{b}^{\ast }log(x))$$

where *y* is the EC_10_ or EC_50_ threshold concentration, x is exposure time in hours, *a* is the initial value, *exp(*−*b* * *log(x))* is the exponential function and *m* is the concentration at which an infinite exposure time would theoretically yield no effect. Both the gamma and non-linear relationships were fitted using simple least squares procedures via the function optim in R.

## Electronic supplementary material

Supplementary information
