# Supplementary material for: Comparative toxicity of five dispersants to coral larvae
Source: Sci Rep. 2018 Feb 14;8:3043. doi: 10.1038/s41598-018-20709-2 (PMC5812988; doi:10.1038/s41598-018-20709-2)
Supplement: Supplementary file 1 — Supplementary information [file 41598_2018_20709_MOESM1_ESM.pdf]

Comparative toxicity of five dispersants to coral larvae

Negri, A.P.[1]*, Luter, H.M.[1], Fisher, R.[1], Brinkman, D.L.[1], Irving, P.[2]

[1]Australian Institute of Marine Science, Townsville, QLD, and Perth, WA, Australia

[2]Australian Maritime Safety Authority, Canberra, ACT, Australia

* Corresponding author

email: a.negri@aims.gov.au

Table S-1. Water quality parameters measured at the end of the longest exposure period (24 h). Dissolved oxygen (DO, mg l^{-1}) was measured for each treatment, while pH and salinity were measured for the seawater control and 40 mg l^{-1} treatments.

Treatment	Concentration (mg dispersant l^{-1})								
	0	0.1	1.25	2.5	5	10	20	40	80
DO									
Seawater	7.6	7.5	7.7	7.2	7.2	7.6	7.4	7.4	7.5
Corexit EC9500A	7.6	7.5	7.6	7.3	7.4	7.5	7.4	7.5	7.5
Slickgone LTSW	7.5	7.5	7.3	7.3	7.2	7.4	7.3	7.4	7.2
Slickgone NS	7.5	7.4	7.1	7.2	7.3	7.6	7.4	7.2	7.1
Ardrox	7.6	7.3	7.4	7.5	7.4	7.4	7.5	7.2	7.1
Finisol	7.6	7.4	7.6	7.6	7.4	7.5	7.5	7.4	7.2
SDS	7.4	7.4	7.5	7.4	7.3	7.4	7.2	7.1	7.1
pH									
Control	8.11							8.15	
Corexit EC9500A	8.17							8.16	
Slickgone LTSW	8.09							8.07	
Slickgone NS	8.11							8.12	
Ardrox	8.08							8.13	
Finisol	8.14							8.14	
SDS	8.06							8.10	
salinity									
Control	34							33	
Corexit EC9500A	34							34	
Slickgone LTSW	35							35	
Slickgone NS	33							34	
Ardrox	34							34	
Finisol	34							34	
SDS	33							33	
